# Effect of Acidic Media on Surface Topography and Color Stability of Two Different Glass Ceramics

**DOI:** 10.1055/s-0044-1786842

**Published:** 2024-05-22

**Authors:** Fatma Makkeyah, Nesrine A. Elsahn, Mahmoud M. Bakr, Mahmoud Al Ankily

**Affiliations:** 1 Fixed Prosthodontics Department, Faculty of Dentistry, The British University in Egypt, Cairo, Egypt; 2 Department of Clinical Sciences, College of Dentistry, Ajman University, Ajman, United Arab Emirates; 3 Center of Medical and Bio-allied Health Sciences Research, Ajman University, Ajman, United Arab Emirates; 4 Department of Operative Dentistry, Faculty of Dentistry, Cairo University, Cairo, Egypt; 5 General Dental Practice, School of Medicine and Dentistry, Griffith University, Gold Coast, Queensland, Australia; 6 Oral Biology Department, Faculty of Dentistry, The British University in Egypt, Cairo, Egypt

**Keywords:** acidic media, glass ceramics, surface roughness, color stability

## Abstract

**Objective** The aim of this study was to determine the effect of two acidic beverages (orange juice and H _3_ PO _4_ -containing fizzy drink) on the surface topography and color stability of two commonly used computer-aided design/computer-aided manufacturing (CAD/CAM) ceramic materials.

**Materials and Methods** Sixty samples of two CAD/CAM ceramic materials, lithium disilicate (IPS e-max CAD) and zirconia reinforced lithium silicate (Vita Suprinity), were prepared according to the manufacturer's instructions. The samples were immersed in one of three media (artificial saliva, orange juice and H _3_ PO _4_ -containing fizzy drink) and then stored in an incubator at 37 °C for 24 hours). Before and after immersion in different media, the surface roughness (Ra) of the samples was assessed using profilometer (JITAI8101 Surface Roughness Tester—Beijing Jitai Tech Detection Device Co. Ltd, China) and the color parameters were measured using VITA Easyshade Advance 4.01 (VITA shade, VITA made, VITA). Surface topography was observed using scanning electron microscope (SEM) and surface mineral content was compared before and after immersion. Paired sample *t* -test was used to determine the change in Ra before and after immersion. Two-way analysis of variance was used to determine the effect of different CAD/CAM materials and immersion media on the mean ∆Ra and mean ∆E of the studied groups. Tukey's honest significant difference posthoc test was used for multiple comparisons at a level of significance (α = 0.05).

**Results** A significant increase in Ra and a decrease in the color stability of the two investigated ceramic materials were detected after immersion in the acidic media than in artificial saliva. SEM and energy-dispersive X-ray results revealed the dissolution of the glassy matrix and the exposure of silicate crystals.

**Conclusion** The surface topography and color stability of glass ceramics are affected by the pH of different acidic media.

## Introduction

The oral cavity can be considered an aggressive environment where the natural tooth surface and dental restorations are exposed to an enormous number of factors affecting their properties and characteristics. Of these factors are acidic beverages that can cause erosion resulting in changes in the surface roughness (Ra) and color of different structures. These acidic beverages have various degrees of resistance against the salivary buffering action and can lower the pH of the oral environment causing demineralization and enamel weakening. 1 2 3

Dental erosion is considered a slow-progressing chemically induced loss of hard tooth structures as a result of frequent exposure to nonbacterial acids. 4 Intrinsic and extrinsic factors are involved in the process. Intrinsic factors include bulimia nervosa or gastroesophageal reflux disease. 4 5 6 Extrinsic factors include acidic soft drinks, juices, vinegar-based dressings for salad products, and acidic snacks. 4 7 In a recent review, the prevalence estimates of tooth surface loss due to erosion were found to be 45% for children aged 3 to 6 years and between 20 and 45% for adults. 8 Worldwide reports indicate a rise in usage of carbonated beverages. According to reports, each person consumes 162 L of soft drinks annually. 9

Nowadays, a wide variety of dental materials can be used to restore lost tooth structure: glass-ceramics, hybrid ceramics, polycrystalline zirconia, and resin composites. These materials can be provided as preprocessed blocks to be milled by computer-aided design/computer-aided manufacturing (CAD/CAM) machines to lessen the possibility of lab issues during the manufacturing of indirect restorations. 10 11 In the oral environment, ceramic restorations are exposed to acidic agents that lead to different responses due to different microstructures of the available materials. The esthetics and longevity of this ceramic material are significantly impacted by any changes in Ra. 12 13 Color and light reflection are negatively impacted by a rough surface as the optical reflection decreases with increasing Ra. 14 These impacts of erosive media have not been thoroughly studied yet. This study aimed to investigate the effect of two acidic media (orange juice and fizzy drinks containing H _3_ PO _4_ ) on the Ra and color stability of two widely used CAD/CAM materials.

The null hypothesis is that the investigated beverages have no effect on the Ra or color stability of the investigated CAD/CAM ceramic materials.

## Materials and Methods

### Samples Preparation

A total of 60 disc-shaped samples measuring 10 mm diameter and 2 mm thickness were fabricated from two CAD/CAM materials (30 samples each); lithium disilicate glass-ceramic (IPS e-max CAD; Ivoclar Vivadent, Liechtenstein) and zirconia reinforced lithium silicate glass-ceramic (VITA SUPRINITY PC, VITA Zahnfabrik H. Rauter GmbH & Co. KG. Postfach 1338 D-79704 Bad Säckingen. Germany). The sample size was determined using Minitab statistical software at 95% confidence level and α = 0.5. For each material, 10 cylinders were milled using 5 axis milling machine VHF S1 (Vhf camfacture AG Lettenstraße 10, 72119 Ammerbuch, Germany). Then the cylinders were sawed using the IsoMet 4000 Linear Precision Saw (IsoMet 4000, Buehler, 41 Waukegan Rd. Lake Bluff, IL, USA) to obtain ceramic discs of 2 mm thickness. The discs were cut under integrated coolant delivery system that flooded the samples from both sides of the blade while tracking with blade movement. Finally, the discs were finished, polished, and glazed.

Before assessment, all the samples were immersed in 20 mL of artificial saliva for 24 hours. The following ingredients were dissolved in 1 L of deionized water to obtain artificial saliva: Xanthan gum (0.92), KCl (1.2), NaCl (0.85), MgCl _2_ (0.05), NaH _2_ PO _4_ (0.13), C _8_ H _8_ O _3_ (0.13), and CaCl _2_ (0.13). 10 15 Then, the samples were removed from the artificial saliva, and dried and baseline Ra and color measurements were performed.

The Ra was assessed using profilometer (JITAI8101 Surface Roughness Tester—Beijing Jitai Tech Detection Device Co. Ltd, China). Each sample was measured three times at different areas (in the middle and sides) and the average was calculated to determine the mean values of the Ra in accordance with ISO 11562 recommendations for standardization. 16 17 18

The color of each sample was assessed using the digital spectrophotometer VITA Easyshade Advance 4.01 (VITA shade, VITA made, VITA). The three-dimensional parameters of the color were recorded numerically; L*, a*, and b* values, where L was the axis of lightness, a was the value representing the axes of chromaticity (green-red), and b was the value representing the axes of color (blue-yellow).

### Erosive Media

The samples of each material were divided into three groups according to the erosive media (10 samples each): (i) artificial saliva (as a control group), groups ES (e-max/saliva) and SS (Suprinity/saliva); (ii) fresh orange juice, groups EO (e-max/orange juice) and SO (Suprinity/orange juice); and (iii) H _3_ PO _4_ -containing fizzy drink (PepsiCo, Inc. Purchase, Harrison, New York, United States) groups EP (e-max/Pepsi) and SP (Suprinity/Pepsi).

Before the insertion of the samples, the original pH of these media was measured using pH meter (Jenway 3510 Standard Digital pH Meter, Cole-Parmer North America 625 East Bunker Court Vernon Hills, IL 60061, USA). The pH of the different immersion media was artificial saliva = 7, orange juice = 3.7, and Pepsi = 2.5.

The samples were immersed into 100 mL of each medium in glass bottles. The bottles were kept in an incubator (Model B 28, BINDER GmbH) at 37 °C for 24 hours. 10 19 20 After that all samples were ultrasonically cleaned for 10 minutes in distilled water and then air-dried. According to previous studies, this erosive procedure is comparable to 2.5 years of clinical exposure to acids where teeth are exposed to acids three times per day, 30 seconds/exposure. 21 22 The pH of the media was rechecked at the end of the immersion time and no change was found.

### Measurements

After immersion in different media, the Ra and color parameters were reassessed following the same previous criteria. The changes in Ra and color were calculated. The color change (∆E) was calculated according to the following formula:

ΔE _2-1_ = ([ΔL] ^2^ + [Δa] ^2^ + [Δb] ^2^ ) ^1/2^

### Scanning electron microscope (SEM – EDX)

One sample of each group was examined by SEM (ThermoFisher (USA) Quattro S Felid Emission Gun, Environmental SEM “FEG ESEM”) at the Nanotechnology Research Center at The British University in Egypt to evaluate the surface topography and was analyzed using energy-dispersive X-ray (EDX) at two different points to determine changes in surface chemical composition.

### Statistical Analysis

Statistical analysis of the obtained data was performed using SPSS for Windows (version 26.0; SPSS Inc., Chicago, Illinois, United States). The normal distribution of all variables was checked and verified using Kolmogorov–Smirnov and Shapiro–Wilk tests. The data were found to be normally distributed. Paired sample *t* -test was used to determine the change in Ra before and after immersion. Two-way analysis of variance (ANOVA) was used to determine the effect of different CAD/CAM materials and immersion media on the mean ∆Ra and mean ∆E of the studied groups. Tukey's honest significant difference posthoc test was used for multiple comparisons at a level of significance (α = 0.05).

## Results

### Scanning Electron Microscope

**Fig. 1:**
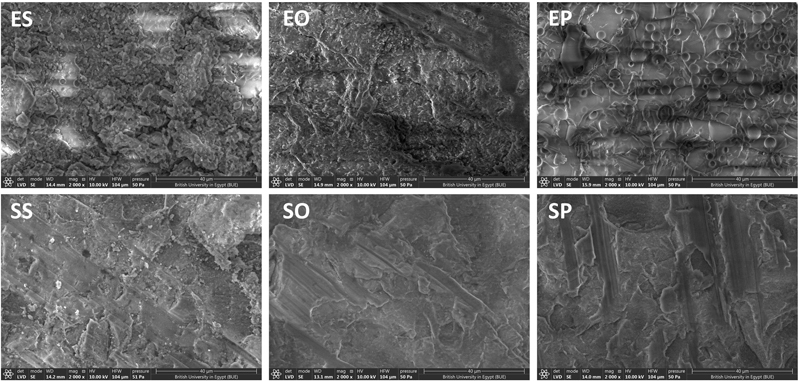
Surface topography of ceramic materials in different erosive media at 2000x magnifications. EO, e-max orange; EP, e-max Pepsi; ES, e-max saliva; SO, Suprinity orange; SP, Suprinity Pepsi; SS, Suprinity saliva.

**Fig. 2:**
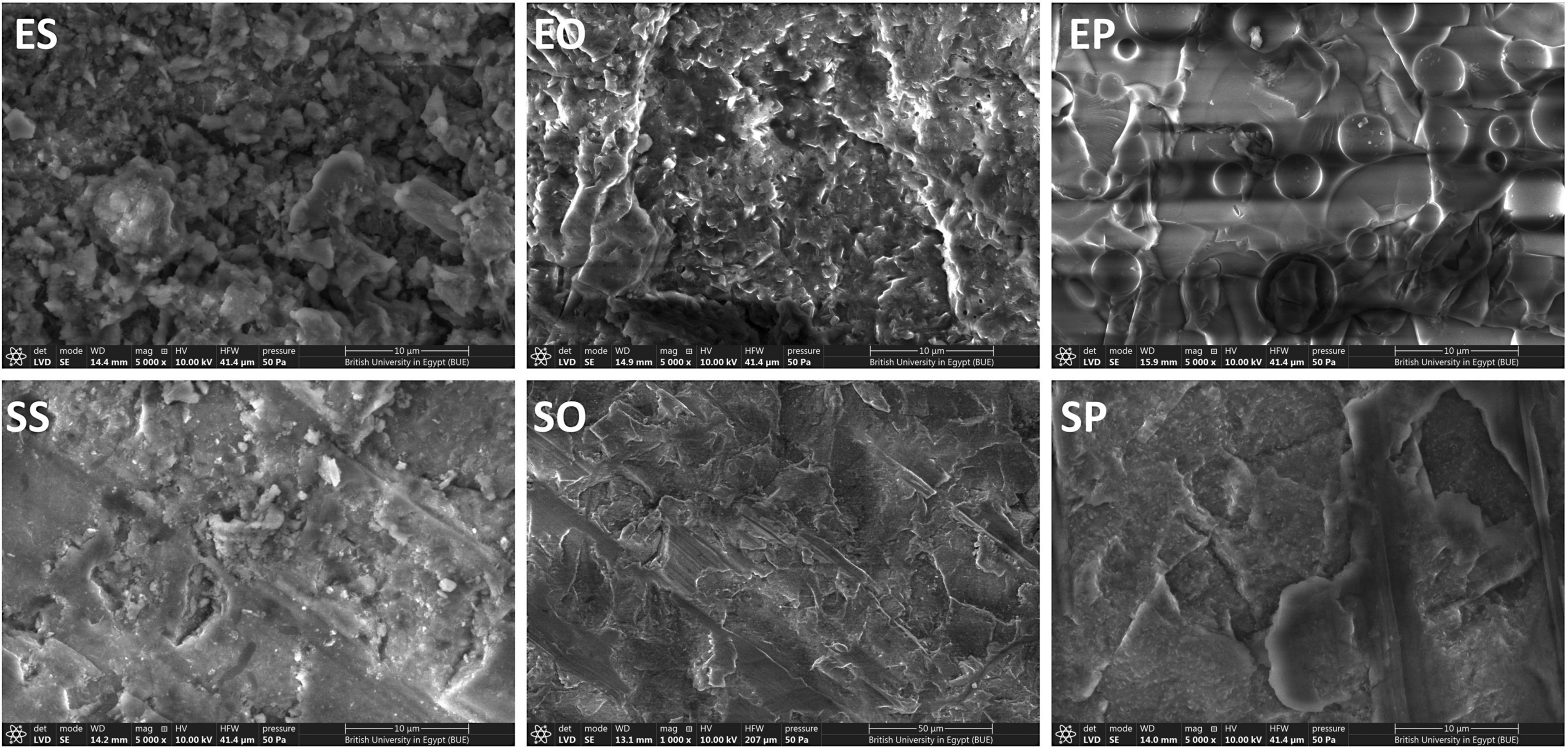
Surface topography of ceramic materials in different erosive media at 5000x magnifications. EO, e-max orange; EP, e-max Pepsi; ES, e-max saliva; SO, Suprinity orange; SP, Suprinity Pepsi; SS, Suprinity saliva.

Surface topography images of the studied specimens at a magnification of 2000X and 5000X are presented in Figs. 1 and 2 . The surface topography of the ES sample showed a plain and a relatively homogeneous surface with normal architecture, and the SS sample showed relatively small roughness with white spots. However, EO showed a relatively rough surface with deep areas that may be due to the removal of the glass matrix, while the SO sample showed rougher surface textures with a wavy curved pattern. The EP sample showed bubble-like regular spherical-shaped roughness affecting the glass matrix and the SP sample showed relatively large dark heterogenous pitted areas.

**Fig. 3:**
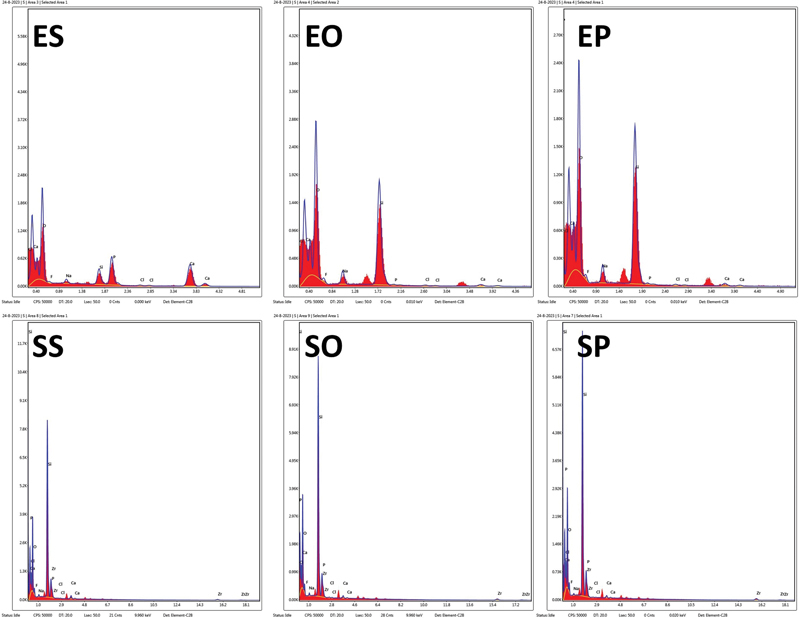
EDX graph of Selected Elements in different groups. EO, e-max orange; EP, e-max Pepsi; ES, e-max saliva; SO, Suprinity orange; SP, Suprinity Pepsi; SS, Suprinity saliva.

Table 1 shows the weight % of different surface components of both materials after immersion in the three investigated media. For both materials, the calcium content was reduced in both the orange juice and Pepsi representing the chelating effect of these acids. Also, EDX analysis graph of selected elements in different groups is presented in Fig. 3 .

**Table 1:** EDX results showing weight% of different surface components of both materials

	IPS e-max CAD			Suprinity		
	Saliva	Orange-Juice	Pepsi	Saliva	Orange-Juice	Pepsi
Oxides	36.37	46.84	43.095	39.87	44.89	44.475
Silica (Si)	26.74	43.815	45.3	26.65	34.435	33.525
Phosphorous (P)	15.35	1.83	0.67	12.365	1.86	2.13
Calcium (Ca)	38.59	2.755	3.48	18.26	0.765	1.715
Zirconia (Zr)				8.995	9.16	9.39

Abbreviations: CAD, computer-aided design; EDX, energy-dispersive X-ray.

Paired sample *t* -test showed that the mean values of Ra were significantly different before and after immersion for all groups; for the groups ES, EO, SS, and SP, *p* -value was less than 0.001, for group EP, *p* -value was 0.011, and for group SO, *p* -value was 0.033.

**Fig. 4:**
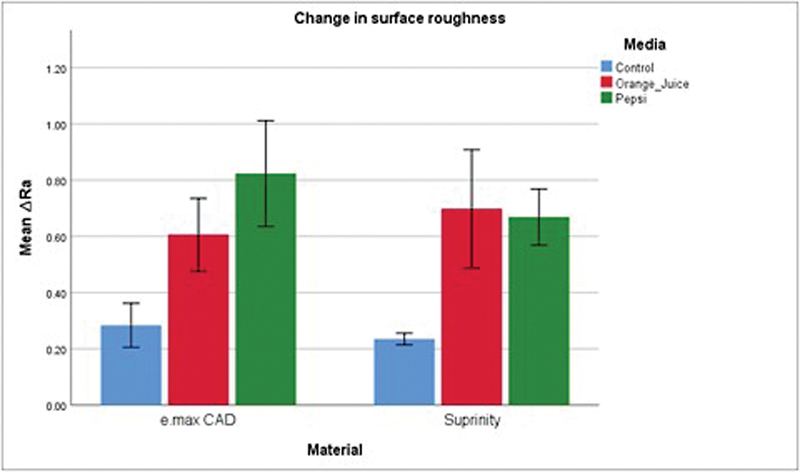
Graph representing changes in surface roughness (Ra). CAD, computer-aided design.

**Fig. 5:**
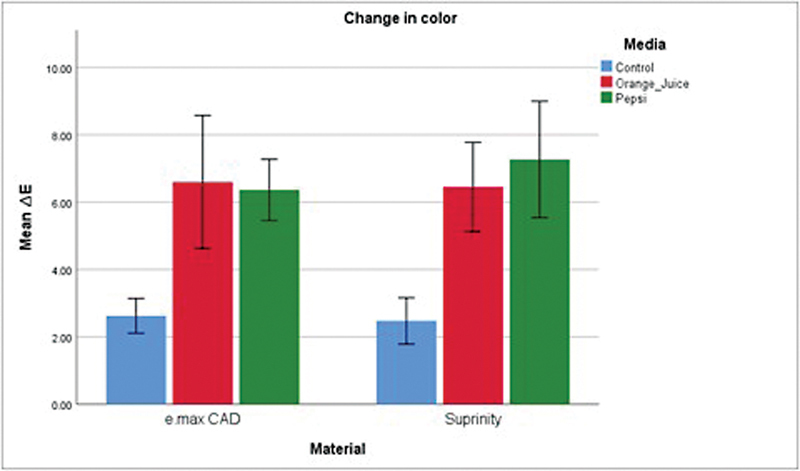
Graph representing changes in color. CAD, computer-aided design.

Descriptive statistics of the mean ∆Ra and mean ∆E are presented in Figs. 4 and 5 , respectively.

Two-way ANOVA showed that the independent variable material ( *p* = 0.456) and the interaction between the material and the erosive media ( *p* = 0.133) had no significant effect on the change in Ra; however, the erosive media was significant in the change ( *p* < 0.001). For both materials, the orange juice group and the Pepsi group showed significant difference from the control group ( *p* < 0.001) and were not significantly different from each other ( *p* = 0.276).

Similarly, for the color change the independent variable material ( *p* = 0.668) and the interaction between the material and the erosive media ( *p* = 0.577) had no significant effect; however, the erosive media was significant in the change ( *p* < 0.001). For both materials, the orange juice group and the Pepsi group showed significant difference from the control group ( *p* < 0.001) and were not significantly different from each other ( *p* = 0.866).

## Discussion

In modern dentistry, CAD/CAM glass ceramics are widely used for the construction of indirect restorations. In the oral cavity, these restorations are subjected to numerous factors affecting their properties, for example, daily brushing, temperature change, change in acid-base levels, and clinical dental procedures. The daily exposure of teeth to different acidic beverages leads to unfavorable changes. According to Statista, the average volume per person consumption of nonalcoholic beverages is expected to be 128.2 L in 2024 and anticipated to reach 159.1 L by 2027. 23 Orange juice and Pepsi selected in this study were the most commonly consumed acidic beverages.

This study aimed to investigate the changes of Ra and color of two types of commonly used dental ceramic CAD/CAM materials after being immersed in acidic agents (orange juice and H _3_ PO _4_ -containing fizzy drink) for 24 hours. The erosive media used significantly affected the Ra and color of the investigated materials. Thus, the null hypothesis was rejected. The results of Ra values demonstrated that the ceramics evaluated cannot be considered as chemically inert. The Ra was increased significantly in all media; however, acidic media showed corrosive effects on the investigated materials. The corrosive degradation was previously explained by two mechanisms. 24 It starts with leaching of alkali ions from the ceramic surface due to the penetration of the hydrogen ions from the aqueous environment into the ceramic, followed by the dissolution of the silicate network (Si-O-Si) of the ceramic. These observations have been previously confirmed by SEM. 24 25 Generally, alkali metal ions are less stable in the glass phase than in the crystalline phase and thus will be leached more rapidly. 24 The release of silicone ions and other ions such as potassium and sodium from the surface of the ceramic can cause porosities in the glass matrix. 26 In this study, SEM photomicrographs and Ra values confirm these mechanisms. The EDX measurements showed an increase in the silicate weight% for both materials and decrease in the calcium and phosphorous wt%; these findings may be attributed to a higher rate of dissolution of the glassy phase than the dispersed lithium disilicate crystals. However, the zirconia wt% of the zirconia-reinforced lithium silicate ceramic was not affected suggesting the resistance of zirconia to such erosive effect. Ra of 0.2 µm is the clinically accepted limit for rough surface above which increased bacterial retention can be detected 10 and Ra values between 0.2 and 0.5 µm can be perceived in some patients by their tongue. 27 Similarly, Al Wadei in 2023 28 reported an increase in Ra and color change after the immersion in different staining materials of green tea, coffee, and Coca-Cola. This finding aligns with previous studies that showed a considerable increase in CAD/CAM lithium disilicate and feldspathic ceramic roughness upon exposure to high temperatures and low pH of acidic liquids. 29 30 31 32

Color stability is considered to be an important aspect in the success or failure of any restoration. 33 A positive correlation has been detected in earlier researches examining the relationship between surface topography and color stability of different dental restorations. 13 17 34 35 36 37 38 The color stability is influenced by the amount of reflection and scattering of light as rough surfaces are more prone to discoloration because of its elevated stain retention capacity. 39 40 41

*In vitro* studies revealed that the detectable and clinically acceptable limits of ∆E were 2.8 and 4.2 units, respectively. 34 42 The results of color change in this study were found to be below the detectable threshold for the saliva groups; however, the groups of erosive media showed a significant color change that was above the clinically accepted threshold. According to Alsilani et al, exposure duration, solution type, and material composition are primary factors that determine the amount of color change. 33 The findings of this study are in accordance with previous studies that reported deleterious effect of acidic agents on dental ceramics 43 44 and with Alp and Suba, 45 who reported that the color stability of Vita Suprinity was affected by citric acid (pH 2) more than artificial saliva. The crystalline phase of dental ceramics is significantly more stable than the glassy phase, which leads to the selective release of alkaline ions that leads to this situation causing increased roughness of the exposed surface, rise in plaque accumulation, wear of tooth or antagonist materials, and discoloration of restorations.

The aim of this study was to determine the erosive effect of the investigated beverages on the properties of the restorative material. A limitation of the current work could be that the erosive protocols applied may not precisely predict material behavior intraorally. In the oral environment, there is a dynamic change in pH due to the buffering capacity of saliva that could not be applied in the erosive model of this *in vitro* study. Further long-term *in vivo* studies are recommended not only to determine the survival of CAD-CAM materials in various erosive media but also to determine ways to counteract and minimize the erosive effect of such beverages on both hard dental tissue and indirect restorative materials are recommended.

## Conclusion

Commonly used acidic beverages can affect Ra and color stability of different types of glass ceramics.
